# Research progress on the relationship between the TdT gene and autoimmune diseases

**DOI:** 10.3389/fimmu.2026.1938776

**Published:** 2026-09-08

**Authors:** Yangyang Man, Xueli Yang, Xiaoni Chen, Yi Liu, Jiahua Hu, Shaofeng Jiang, Xianliang Hou

**Affiliations:** 1 Laboratory Center, Guangxi Key Laboratory of Metabolic Reprogramming and Intelligent Medical Engineering for Chronic Diseases, The Second Affiliated Hospital of Guilin Medical University, Guilin, China; 2 Department of Central Laboratory, Shenzhen Hospital, Beijing University of Chinese Medicine, Shenzhen, Guangdong, China; 3 Guangxi Key Laboratory of Tumor Immunology and Microenvironmental Regulation, School of Basic Medicine Sciences, Guilin Medical University, Guilin, China

**Keywords:** autoimmune diseases, immune tolerance, immunity, TdT gene, V(D)J recombination

## Abstract

Autoimmune diseases (AIDs) are caused by defects in immune tolerance or abnormalities in immune regulation, leading to damage to host organs. The pathogenesis of AIDs is complex, and their clinical manifestations are diverse. Currently, the pathogenesis of AIDs is not fully understood. Accumulating evidence from experimental models suggests that TdT-mediated antigen receptor diversification may influence autoimmune responses and immune regulation. Although emerging evidence from human autoimmune diseases has been reported, the current understanding of the relationship between TdT and AIDs remains limited. Understanding the mechanisms by which TdT influences autoimmune responses may provide new insights into disease pathogenesis and future therapeutic exploration. This review summarizes relevant information on the TdT gene and discusses current evidence regarding the relationship between TdT and AIDs, with particular emphasis on findings from experimental models and emerging evidence from human autoimmune diseases.

## 1 Introduction

AIDs are diseases caused by the disorder of the body’s immune system that attacks the host itself (1). They are a class of diseases in which the immune tolerance to self-components is broken, leading to attacks on the body’s own organs, tissues, or cells, resulting in damage (2). AIDs are a class of multifactorial disorders that include, for example, rheumatoid arthritis (RA), systemic lupus erythematosus (SLE), and Sjögren’s syndrome (SS) (3). In recent years, the prevalence of AIDs has been increasing, making them one of the major diseases threatening human health (4). Currently available immunomodulatory therapies have substantially improved the management of autoimmune diseases. Although some therapies may exert broad immunomodulatory effects, many are designed to target specific immune pathways and are selected based on disease mechanisms. However, treatment responses vary among patients, and some therapies may be associated with adverse effects, including an increased risk of infection and other treatment-related complications (5, 6). Despite these therapeutic advances, the mechanisms underlying AIDs remain incompletely understood (7), highlighting the need for continued investigation of more precise and disease-specific therapeutic strategies.

Among the mechanisms potentially contributing to autoimmune responses, antigen receptor diversification is of particular interest. Terminal deoxynucleotidyl transferase (TdT) is a key enzyme involved in V(D)J recombination and increases B-cell and T-cell receptor diversity by mediating junctional diversification during antigen receptor rearrangement (8). Theoretically, enhanced junctional diversity may increase the probability of generating autoreactive lymphocyte receptors, thereby influencing immune tolerance and contributing to autoimmune responses (9). Conversely, reduced TdT activity or genetic deficiency may restrict antigen receptor diversity and has been associated with attenuated autoimmune manifestations in experimental models (10–12).

Previous studies have shown that adult mice deficient in the TdT gene exhibit competent immune responses (13, 14). Experimental studies have further demonstrated that TdT deficiency is associated with reduced autoimmune manifestations in several disease models. In lupus-prone mouse models, including NZB × NZW F1 and MRL/lpr mice, TdT deficiency reduced autoimmune nephritis in NZB × NZW F1 mice and decreased autoimmune disease severity in MRL/lpr mice (10, 11). In addition, TdT deficiency reduced the incidence of autoimmune diabetes and pancreatic infiltration in NOD mice (12). In human rheumatoid arthritis, TdT expression has also been detected in synovial tissues, although the available evidence remains limited and does not establish a direct pathogenic role (15). These findings provide further support for investigating the potential relationship between TdT-mediated antigen receptor diversification and autoimmune disease. Based on these observations, this review summarizes the current evidence regarding the relationship between the TdT gene and AIDs, with particular emphasis on findings from experimental models and emerging evidence from human autoimmune diseases.

## 2 TdT gene

### 2.1 Overview of the TdT gene

The TdT gene (also known as the Dntt gene) is a member of the DNA polymerase X family and encodes a template-independent DNA polymerase, TdT, which catalyzes the addition of deoxynucleotides to the 3’-hydroxyl terminus of oligonucleotide primers (16, 17). In 1960, TdT was first discovered in calf thymus (18). TdT exists in two major isoforms, a short isoform of approximately 58 kDa and a long isoform of approximately 66 kDa (19, 20). Through its role in antigen receptor junctional diversification, TdT contributes to B-cell and T-cell receptor repertoire diversity, providing a potential mechanistic link between antigen receptor diversification and autoimmune responses.

### 2.2 Expression and function of the TdT gene

The expression of TdT is tissue-specific and restricted to lymphoid precursors, playing an important role in the development and function of the immune system (21). Deibel et al. (22) examined TdT expression in the human thymus during ontogeny. In five fetal thymus samples, the activity of this enzyme was almost undetectable. At birth, terminal transferase activity remained very low. Maximal expression of enzyme activity occurred between 10 and 40 months of age.

TdT catalyzes the addition of non-templated nucleotides (N nucleotides) at the junctions of rearranging immunoglobulin heavy- and light-chain genes and T-cell receptor genes during V(D)J recombination, thereby contributing to antigen receptor junctional diversity (8, 14, 23). This is largely because the amount of TdT expressed in lymphocyte precursors correlates with the degree of N-region diversity in antigen receptors (24, 25). In addition, studies have confirmed that after introducing a disruptive mutation into the TdT gene, TdT-deficient mice show an almost complete absence of insertional junctions in Ig heavy chain and TCR sequences (14). Therefore, TdT is a key factor influencing the outcome of V(D)J recombination (8). Although recombination does not require TdT, it increases the junctional diversity of Ig and TCR genes, thereby increasing lymphocyte receptor diversity. During V(D)J recombination, TdT-mediated nucleotide addition contributes to junctional diversity, while the RAG1/RAG2 recombinase complex initiates antigen receptor gene rearrangement and diversification (26, 27). Furthermore, researchers have also demonstrated that stable and progressive upregulation of TdT defines the lymphoid developmental trajectory (28).

Furthermore, in the mouse thymus, two alternatively spliced forms of TdT mRNA have been identified: TdTL mRNA (the long form of TdT isolated from BALB/c mice) and TdTS mRNA (the short form of TdT isolated from BALB/c mice) (29). The corresponding 20-amino-acid insertion found near the carboxyl terminus of TdTL significantly alters the function of the enzyme; unlike TdTS, TdTL is unable to catalyze N-region insertions at the recombination junctions of V(D)J site-specific recombination substrates (30). The potential link between TdT-mediated antigen receptor diversification and autoimmune responses is illustrated in **Figure 1**.

**Figure 1:**
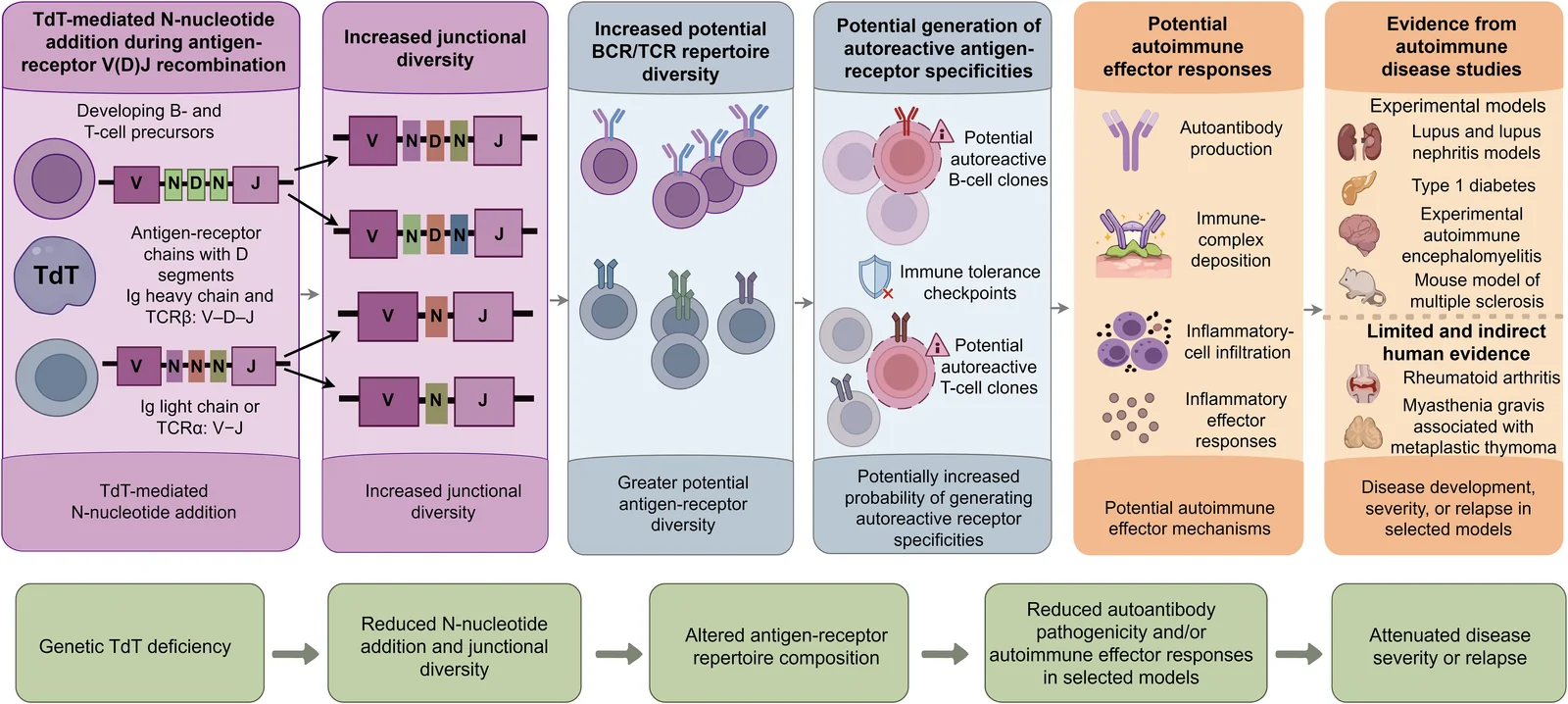
Proposed mechanism underlying the involvement of TdT in autoimmune diseases. TdT mediates N-nucleotide addition during antigen receptor V(D)J recombination, thereby increasing junctional diversity of immunoglobulin and T-cell receptor genes and contributing to B-cell receptor (BCR) and T-cell receptor (TCR) repertoire diversification. Increased antigen receptor diversity may increase the potential generation of autoreactive receptor specificities, which may contribute to autoimmune effector responses, including autoantibody production, immune-complex deposition, inflammatory cell infiltration, and tissue injury. Genetic deficiency of TdT reduces N-nucleotide addition and alters antigen receptor repertoire composition, which has been associated with attenuated autoimmune responses and reduced disease severity or relapse in experimental autoimmune models. Current evidence supporting this mechanism is mainly derived from animal studies, whereas evidence from human autoimmune diseases remains limited.

### 2.3 Chromosomal localization of the TdT gene

Studies on the chromosomal localization of the TdT gene have primarily focused on humans and mice. This gene plays an important role in the development of the immune system, particularly in the early stages of B-cell and T-cell development, by increasing N-region diversity during V(D)J gene recombination, thereby promoting the generation of antibody and T cell receptor diversity.

Previous studies have clarified the chromosomal location of the human TdT gene (31, 32). Isobe et al. (31) used complementary DNA clones to screen mouse × human somatic cell hybrid DNA and, through Southern blot analysis, determined that the human TdT gene is located on chromosome 10, specifically in the region 10q23-q25. Subsequently, Yang-Feng et al. (32) precisely mapped the human TdT gene to the q23→q24 region of chromosome 10 using *in situ* hybridization and Southern blot analysis. Breakages in these regions are associated with certain diseases (32). In addition, chromosomal localization studies of the mouse TdT gene have shown that the gene is located on mouse chromosome 19 (32). A molecular genetic linkage map of mouse chromosome 19, including the lpr, Ly-44, and TdT genes, has been constructed (33).

### 2.4 Regulation of TdT gene expression

Studies have shown that the expression of the TdT gene is regulated at the transcriptional level (34). A novel cis-regulatory element in the 5’ region of the mouse TdT locus has been identified, which may play an important role in the complex regulation of the TdT gene (34).

Traditionally, the core promoter of TdT is thought to contain D, D’, the initiator (Inr), and the downstream basal element (DBE). However, the combination of these elements does not fully explain the complex regulatory pattern of TdT expression (34). Studies have shown that, in addition to the core promoter region, the flanking regions of the TdT gene also contain important cis-regulatory elements. For example, the promoter of the C1 inhibitor gene contains a purine- and pyrimidine-rich fragment that can enhance transcriptional activity (35). This observation suggests that similar sequences in the TdT gene may also affect its transcription.

Furthermore, TdT-interacting factor 1 (TdIF1) was initially identified as a protein that directly binds to the DNA polymerase TdT, and it can also bind to the transcription factor TReP-132 as well as the histone deacetylases HDAC1 and HDAC2 (36). These findings suggest that TdIF1 plays a role in transcriptional regulation. The study by Takashi Kubota et al. (36) further confirmed that TdIF1 can recognize specific DNA sequences and regulate gene transcription through its helix-turn-helix (HTH) and AT-hook domains.

Furthermore, the accessibility of chromatin directly affects the binding of transcription factors to DNA. Histone acetylation is generally associated with gene activation, whereas deacetylation is associated with gene silencing. The interaction of TdIF1 with the histone deacetylases HDAC1 and HDAC2 suggests that the transcription of the TdT gene may be regulated by chromatin modifications (36). HDAC1 and HDAC2 are histone deacetylases involved in catalyzing the removal of acetyl groups from histones, leading to a more compact chromatin structure, which is generally associated with transcriptional repression (37). Therefore, the binding of TdIF1 to HDAC1/HDAC2 may mediate histone deacetylation in the promoter region of the TdT gene, thereby repressing the transcription of the TdT gene (38).

## 3 Relationship between TdT and AIDs

### 3.1 TdT and rheumatoid arthritis

In RA, local antigen receptor rearrangement and receptor revision have been investigated as potential processes associated with autoreactive antibody responses in the synovium (15).

Zhang et al. (15) used reverse transcription-polymerase chain reaction (RT-PCR) to detect recombination-activating gene (RAG) and TdT transcripts in unselected and B-cell-enriched synovial and peripheral blood mononuclear cells from 12 patients with RA. They found evidence of RAG-mediated secondary immunoglobulin κ (Ig κ) light-chain rearrangement in the synovial tissues of approximately one-third of the patients. TdT mRNA was detected in 6 of 12 synovial samples; however, TdT expression did not consistently coincide with RAG expression patterns across the examined samples. Therefore, the original study did not demonstrate a clear association between TdT and RAG expression in RA synovial tissues.

Subsequently, to test the hypothesis that RAG-dependent revision of immunoglobulin genes in germinal centers may contribute to local autoantibody production in Hashimoto’s thyroiditis (HT) and RA, Sening et al. (39) examined the expression of RAG and TdT in HT and RA tissues *in situ*. They detected TdT and RAG expression in extrafollicular lymphocytes of control tonsil sections. In contrast, only rare TdT-expressing cells were identified in 11 of 19 HT samples and 2 of 20 RA samples. Germinal center B cells were consistently negative for TdT and RAG. These results indicate that local RAG-dependent receptor revision in germinal centers is unlikely to be responsible for autoantibody production in HT and RA. The presence of TdT-positive extrafollicular cells may represent an influx of immature cells under conditions of chronic immune stimulation. In summary, current evidence does not support a direct role for TdT in germinal-center receptor revision or autoantibody production in RA. The presence of rare TdT-positive extrafollicular cells may instead reflect the recruitment of immature lymphocytes under conditions of chronic immune stimulation, and the functional significance of these cells in RA remains to be clarified.

### 3.2 TdT and autoimmune kidney diseases

The potential contribution of TdT-dependent antigen receptor diversity to autoimmune renal injury has been investigated in lupus-prone mouse models (10, 11, 40). Previous studies have shown that in TdT knockout mice (TdT^-/-^), the T-cell repertoire shows reduced peptide specificity; natural autoantibodies, particularly anti-DNA autoantibodies, are less polyreactive and have reduced average affinity. Therefore, inhibition of TdT during early B/T cell development may be involved in controlling autoimmunity. To investigate the effect of TdT inhibition on autoimmune-prone mice, Conde et al. (10) introduced a TdT null mutation into the (NZB × NZW) F1 (B/W) mouse strain. They found that TdT deficiency significantly reduced the incidence of autoimmune nephritis and prolonged survival compared to control mice. Surprisingly, long-term surviving TdT-/- mice produced amounts of anti-DNA and anti-histone autoantibodies similar to those in TdT+/+ mice. However, these TdT-/- mice showed evidence of kidney inflammation, with immune deposits restricted to the mesangium, whereas basement membrane deposits were clearly associated with overt kidney disease. Their findings suggest that TdT deficiency may attenuate autoimmune pathology in this experimental model and provide a rationale for further investigation of TdT-related mechanisms in autoimmunity. In summary, their findings provide a more comprehensive understanding of the mechanisms underlying lupus nephritis.

Feeney et al. (11) further generated TdT-deficient MRL/lpr, Fas-deficient (MRL-Faslpr) mice and found that they had prolonged lifespans, reduced incidence of skin lesions, and greatly decreased serum levels of anti-dsDNA, anti-chromatin, and IgM rheumatoid factor. The systemic hypergammaglobulinemia characteristic of MRL-Faslpr mice was also greatly reduced, as was the proportion of CD4-CD8- B220+ (double-negative) T cells. Notably, although many mice still showed signs of kidney disease at six months, IgG deposition in the kidneys was significantly reduced. Their study indicates that TdT deficiency alleviates renal pathology in autoimmune lupus disease.

In short, TdT may play a key role in autoimmune kidney diseases. N-region diversity in T cells, B cells, or both may be required for the development of more comprehensive and severe autoimmune kidney diseases.

### 3.3 TdT and myasthenia gravis

The thymus plays an important role in T-cell development and immune tolerance and is particularly relevant to thymoma-associated MG. Because TdT is predominantly expressed in immature lymphoid cells and contributes to antigen receptor diversification during early lymphocyte development (8, 21, 22), the presence of TdT-positive immature T cells may reflect altered thymocyte development within the thymic microenvironment. This provides a biological rationale for examining TdT-positive cells in thymoma-associated MG.

Thymoma is well known for its association with paraneoplastic autoimmune diseases such as MG. Only one case of metaplastic thymoma associated with MG had been reported before Tajima et al. described a second case in a 44-year-old woman (41). Although intratumoral TdT-positive immature T cells are generally scarce in metaplastic thymoma, they were abundant in this case. The authors suggested that this unexpected accumulation of immature T cells might be associated with the development of MG. In the previously reported case of metaplastic thymoma associated with MG, the lymphocyte composition was not thoroughly examined; therefore, whether a similar accumulation of TdT-positive cells was present remains unknown (42).

Collectively, these findings suggest that the abnormal accumulation of TdT-positive immature T cells may be associated with MG in specific thymoma-associated settings. However, the available evidence is limited to a small number of case reports, and the exact relationship between TdT-positive immature T cells and MG remains to be clarified.

### 3.4 TdT and experimental autoimmune encephalomyelitis

In EAE, TdT-dependent TCR repertoire diversification has been investigated as a determinant of autoimmune disease progression and relapse (43).

Fazilleau et al. (43) compared the TCRαβ repertoire of myelin oligodendrocyte glycoprotein (MOG)-specific T lymphocytes in C57BL/6 mice and TdT-deficient littermates (TdT-/-) during experimental autoimmune encephalomyelitis (EAE). At disease onset, EAE severity was similar in TdT+/− and TdT−/− mice, and MOG-specific T cells from both groups displayed similar public Vα-Jα and Vβ-Jβ rearrangements. During the subsequent disease course, TdT+/+ and TdT+/− mice experienced successive EAE relapses, whereas TdT−/− mice eventually recovered. During the first relapse in TdT+/− mice, new public Vα-Jα and Vβ-Jβ rearrangements emerged that were distinct from those detected at disease onset. Most of these rearrangements contained N-additions and were detected in CNS-infiltrating T lymphocytes.

These findings indicate that the initial encephalitogenic response can occur independently of TdT-mediated N-region diversification, whereas subsequent EAE relapse depends on TdT-mediated N-region diversification of the autoreactive TCR repertoire. However, further studies are needed to determine how TdT-dependent N-region diversification contributes to the generation of relapse-driving autoreactive T-cell clones and whether this mechanism is also relevant to other autoimmune diseases.

### 3.5 TdT and other AIDs

Molano et al. (40) hypothesized that genetic deletion of TdT would modulate disease in lupus-prone mice. To test this hypothesis, they generated TdT-deficient MRL/lpr mice and found that, compared with TdT+ littermates, TdT− mice had significantly reduced serum levels of anti-dsDNA antibodies and anti-dsDNA-producing splenocytes. Furthermore, TdT- mice also exhibited significantly reduced proteinuria, glomerular IgG deposition, and pathological kidney disease. Therefore, their study demonstrated that genetic deletion of TdT reduces autoantibody production and clinical disease in MRL/lpr mice. The study by Robey et al. (12) further extended the scope of TdT’s role in autoimmunity. They not only studied TdT-deficient MRL/lpr mice but also TdT-deficient non-obese diabetic (NOD) mice. NOD mice are a spontaneous model of type 1 diabetes, the pathogenesis of which involves T cell-mediated destruction of pancreatic islet cells. Robey et al. (12) observed that TdT-deficient NOD mice had a reduced incidence of diabetes and decreased pancreatic infiltration, suggesting that TdT-dependent receptor diversification may influence T-cell autoreactivity. Additionally, they observed that TdT-deficient MRL/lpr mice exhibited alleviated glomerulonephritis and prolonged lifespan, consistent with the findings of Molano et al. (40) and further supporting a potential role for TdT-dependent receptor diversification in lupus pathogenesis. Robey et al. (12) also observed a reduced incidence of autoimmune diseases in TdT-deficient mice, suggesting that TdT deficiency may influence autoimmune susceptibility. Whether this effect is associated with altered autoantibody production remains unclear.

However, these studies still have limitations, as they were conducted primarily in mouse models. Although MRL/lpr mice and NOD mice are commonly used models for studying lupus and type 1 diabetes, they do not fully represent human diseases. Moreover, the role of TdT in human AIDs may be more complex than that in mouse models. Therefore, further studies are needed to validate these findings and explore the specific mechanisms of TdT in human AIDs. Although a broader range of autoimmune diseases was considered in our extended literature search, direct evidence linking TdT or DNTT to additional autoimmune diseases remains limited. Further studies are therefore needed to determine whether TdT-mediated antigen receptor diversification contributes to a broader spectrum of autoimmune diseases. Future research can be carried out in several aspects: first, further investigation of the expression and function of TdT in human AIDs is needed. Second, currently no TdT-targeted therapies have been developed for autoimmune diseases, and the potential effects of modulating TdT activity on autoimmune responses should be further investigated in appropriate experimental models. Finally, the interaction between TdT and other immune regulatory molecules needs to be studied to gain a more comprehensive understanding of the regulatory mechanisms of autoimmune responses. A better understanding of these mechanisms may help clarify the potential clinical relevance of TdT in autoimmune diseases. The major findings regarding the relationship between TdT and autoimmune diseases are summarized in **Table 1**.

**Table 1:** Summary of major studies examining TdT-related findings in autoimmune diseases and experimental models.

Disease or model	Study subjects or samples	TdT-related observation	Major findings	Interpretation and evidence limitations
Rheumatoid arthritis	Synovial tissue and peripheral blood mononuclear cells from 12 patients with RA	TdT mRNA was detected in 6 of 12 synovial samples, but TdT expression did not consistently coincide with RAG expression patterns.	Evidence of RAG-mediated secondary Ig κ light-chain rearrangement was found in the synovium of approximately one-third of the patients	TdT expression may indicate the presence of immature B cells in RA synovial tissue, but its direct involvement in receptor revision or autoantibody production remains uncertain
Hashimoto thyroiditis and rheumatoid arthritis	Tissue samples from 19 patients with Hashimoto thyroiditis and 20 patients with RA	Rare TdT-positive extrafollicular lymphocytes were detected in 11 Hashimoto thyroiditis samples and 2 RA samples; germinal-center B cells were negative for TdT and RAG	No evidence supported RAG-dependent receptor revision within germinal centers	TdT-positive cells may represent an influx of immature lymphocytes under chronic immune stimulation rather than local receptor revision
Lupus nephritis model	TdT-deficient and TdT-sufficient NZB × NZW F1 mice	TdT status or relevant finding	TdT deficiency reduced the incidence of autoimmune nephritis and prolonged survival; anti-DNA and anti-histone antibody levels remained similar in long-term survivors, but renal immune deposits were largely restricted to the mesangium	TdT deficiency may alter the pathogenicity or tissue-deposition pattern of autoantibodies rather than completely preventing their production
Lupus-prone MRL-Fas(lpr) model	TdT-deficient MRL-Fas(lpr) mice	Genetic deletion of TdT	Prolonged survival and reductions in skin lesions, autoantibody levels, hypergammaglobulinemia, and renal IgG deposition were observed	TdT-dependent antigen-receptor diversity may contribute to systemic autoantibody production and lupus-related tissue injury
Lupus-prone MRL/lpr model	TdT-deficient MRL/lpr mice	Genetic deletion of TdT	Anti-dsDNA antibodies, anti-dsDNA-producing splenocytes, proteinuria, glomerular IgG deposition, and renal pathological injury were reduced	TdT deficiency attenuated both autoantibody production and renal disease in lupus-prone mice
Type 1 diabetes and lupus models	TdT-deficient NOD mice and TdT-deficient MRL-Fas(lpr) mice	Genetic deletion of TdT	NOD mice showed reduced diabetes incidence and pancreatic infiltration, whereas MRL-Fas(lpr) mice showed alleviated glomerulonephritis and prolonged survival	TdT-dependent antigen-receptor diversity may contribute to disease development across both T-cell-dominant and autoantibody-associated autoimmune models.
Myasthenia gravis associated with metaplastic thymoma	A 44-year-old woman with metaplastic thymoma and myasthenia gravis	Abundant intratumoral TdT-positive immature T cells were observed	The number of immature T cells was unusually high compared with typical metaplastic thymomas	Abnormal accumulation of immature thymic T cells may be associated with MG, although the evidence is limited to a single case report
Experimental autoimmune encephalomyelitis	MOG-induced EAE in TdT-deficient and TdT-sufficient mice	TdT-deficient mice lacked newly emerging TCR rearrangements containing N-nucleotide additions during relapse	Initial disease severity was similar between groups, but TdT-deficient mice recovered and did not undergo successive relapses	TdT-dependent diversification of the autoreactive TCR repertoire may be particularly important for disease relapse rather than initial disease onset

## 4 Summary and outlook

Current evidence, derived predominantly from experimental animal models, suggests that TdT-mediated antigen receptor diversification may influence autoimmune responses and disease manifestations. However, evidence from human autoimmune diseases remains limited, and the precise contribution of TdT to disease initiation, progression, and relapse has not been fully established. Future studies should further investigate the expression and functional significance of TdT in human autoimmune diseases and clarify how TdT-dependent antigen receptor diversification influences autoreactive lymphocyte repertoires. Such studies may provide a stronger mechanistic basis for evaluating the potential clinical relevance of TdT in autoimmune diseases.
